# Immunological Protein Signature During Acute Exercise

**DOI:** 10.1111/apha.70125

**Published:** 2025-10-29

**Authors:** Charlotte Wenzel, David Walzik, Tiffany Wences, Sina Trebing, Klaus Meyer, Andreas Groll, Philipp Zimmer, Niklas Joisten

**Affiliations:** ^1^ Institute of Sport and Sport Science, Research Group “Sports Medicine”, TU Dortmund University Dortmund Germany; ^2^ Bevital AS Bergen Norway; ^3^ Department of Statistics TU Dortmund University Dortmund Germany

The modulation of the immune system is suspected to be one key mechanism underlying the health benefits of physical exercise. Acute exercise (single bout) triggers transient changes in blood levels of various immunological factors, which are central to the immediate responses and adaptations of the immune system [1]. While many studies have focused on the concentration of individual cytokines before and after acute exercise [2, 3, 4, 5], there is a lack of (i) a high‐resolution examination of the kinetic changes during exercise itself, (ii) an analysis of a comprehensive panel of immunological signature proteins, and (iii) a systematic understanding of dynamic immunological alterations. Here, we investigated a comprehensive immunological protein signature using high‐resolution blood sampling during aerobic exercise.

Twelve healthy adults (*n* = 6 females, *n* = 6 males; age [years]: 25.5 ± 2.7, BMI [kg/m^2^]: 22.9 ± 3.1, V̇O_2_peak [ml·kg^−1^·min^−1^]: 45.4 ± 9.7; values are mean ± SD) performed a 40‐min aerobic exercise session at 60% of their V̇O_2_peak between 8 and 10 AM after an overnight fast. Venous blood samples were collected at baseline, after warm‐up, 10 min, 15 min, 30 min, 40 min (end) as well as in recovery (1h post) and processed immediately after collection. The study was approved by the ethics committee of the Leibniz Research Centre for Working Environment and Human Factors (IfADo, Dortmund, Germany). Detailed information on the participants' characteristics and the study design can be found in Figure 1A and at https://sportsmedicine‐dortmund.shinyapps.io/beat.

Targeted immunological proteome profiling, including cytokines, chemokines, effector molecules, cell surface markers, growth factors, and immune regulators, was performed using the Olink® 96 Immuno‐Oncology Panel (Table S1: Proteins). The final data output consisted of Normalized Protein expressions (NPX) of 92 proteins quantified. In order to assess significant changes over time, linear mixed models (LMMs) were calculated for each protein, with protein expression serving as the dependent variable. Time point and sex were considered as fixed effects, and subject ID as a random effect. Proteins with a Benjamini‐Hochberg adjusted *p*‐value < 0.05 and at least one significant time coefficient were considered for further analysis. Significant proteins were grouped into four clusters based on hierarchical clustering of their log2 fold changes across all time points. A random forest model was trained for each protein concentration at 1 h post exercise (including all proteins previously identified as significant by LMM). To interpret the contribution of exercise‐induced protein changes on their concentration in the recovery phase, we used SHapley Additive exPlanations (SHAP).

**FIGURE 1:**
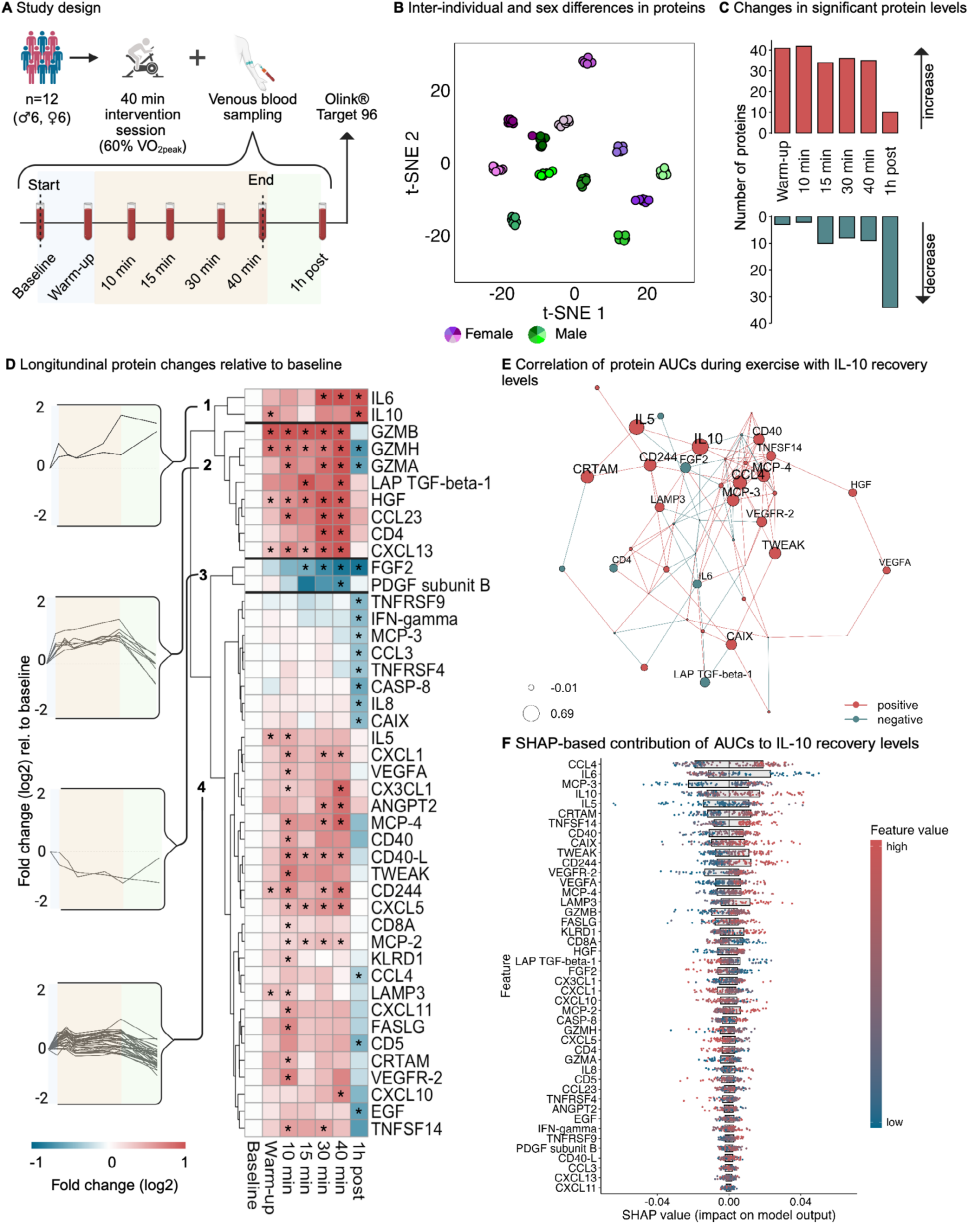
Comprehensive analysis of protein dynamics during acute exercise and training‐induced immunological protein changes related to recovery. (A) Study design. (B) t‐SNE of all samples by ID and sex (perplexity 7, 1000 iterations, θ 0.05). (C) Count of proteins that significantly change relative to baseline over time in response to acute exercise. (D) Heatmap of the *z*‐score‐normalised median log2 fold changes of the significant proteins compared to baseline. *: adj. *p* value < 0.05. (E) Correlation network of protein areas under the curve (AUCs) during exercise with IL‐10 values at 1 h post exercise. (F) Random Forest model with SHAP was used to investigate the influence of protein‐based AUC values during exercise on the IL‐10 concentration during recovery. Input data (X) were *z*‐transformed, while target variables (Y) remained unscaled. Hyperparameter optimisation was performed using Bayesian search, and the best parameters were used for model training. Model validation was conducted using the leave‐one‐out method, and model quality was assessed using the root mean square error (RMSE). The y‐axis shows the features (protein AUCs) sorted by their importance for the prediction of the IL‐10 concentration 1 h post exercise. The x‐axis represents the SHAP values, which indicate how strongly each feature influences the model prediction of IL‐10 – positive values increase the prediction, negative values decrease it. SHAP values show the direction and strength of the influence of individual features on the prediction but do not indicate overall model quality or model error.

The t‐SNE analysis resulted in distinct clusters for each individual and a separation between male and female participants (Figure 1B). These results support previous evidence that immune responses to acute exercise are highly individual and influenced by sex, reflected in different protein concentration patterns [6].

After fitting LMMs for each of the 92 proteins across all time points, 44 proteins (48%) showed significant changes compared to baseline (Figure 1C; Table S1: LMM_results), which grouped into four distinct clusters, indicating related responses to acute exercise (Figure 1D).

Cluster 1 (2 proteins), consisting of interleukin (IL)‐6 and IL‐10, increased during exercise and continued to increase (IL‐10) or remained elevated (IL‐6) during recovery. This high‐resolution approach to quantify protein concentrations enables a data‐driven validation of the IL‐6/IL‐10 dynamics described in the literature. The pro‐inflammatory response of IL‐6 to exercise is well documented [5, 7], and it is hypothesised that IL‐6 mediates anti‐inflammatory effects through the upregulation of IL‐10 [8].

Proteins in cluster 2 (8 proteins) initially increased but decreased to or below baseline levels during recovery. These proteins included several granzymes, which are central effector molecules of cytotoxic immune cells, in particular of natural killer (NK) cells. Acute exercise leads to a significant increase in the number and activity of NK cells, followed by a marked decrease during recovery [9]. Indeed, the activity of granzyme‐releasing NK cells largely corresponds to the kinetics of granzyme levels after acute exercise observed in our data.

In cluster 3 (2 proteins), the exercise‐induced decrease in fibroblast growth factor 2 (FGF2) levels is in line with results from a previous study [10]. In contrast to many other growth factors (e.g., vascular endothelial growth factor (Figure 1D) or brain‐derived neurotrophic factor [11]) that increase in response to acute exercise, we observed a decrease of both FGF2 and platelet‐derived growth factor B (PDGF‐B) below baseline levels in an exercise duration‐dependent manner.

Cluster 4 comprises the largest number of proteins (32 proteins), which were relatively close to the baseline concentration during exercise and mainly decreased during recovery. A well‐described cytokine in cluster 4 with a heterogeneous response to acute exercise is interferon gamma (IFN‐γ), which plays a key role in immune regulation, inflammation, and muscular adaptation. IFN‐γ levels can display both an increase and a decrease after acute exercise [12]. Our data extend this observation by showing that IFN‐γ concentration falls below baseline levels already 15 min after the onset of acute exercise.

Altogether, hierarchical clustering emphasizes the relevance of high‐resolution measurements as they reveal different kinetic patterns. While most significantly altered proteins increased (e.g., IL‐6 and IL‐10) during exercise and only a few decreased (e.g., FGF2 and PDGF‐B), this trend was reversed during recovery, with many of the initially increased proteins decreasing below baseline (Figure 1C).

We used correlation network analyses (Figure 1E) and the SHAP algorithm of random forest models (Figure 1F) to identify relevant exercise‐induced changes in protein concentrations (44 protein AUCs as a measure of the total absolute concentration changes) that influence concentrations 1 h post exercise. Importantly, these approaches provide unbiased insights into linear (network analysis) and non‐linear (random forest model in combination with SHAP analysis) interactions between the temporal dynamics of immunological proteins induced by acute exercise.

Considering the results for IL‐10 (Figure 1E,F), C‐C motif chemokine ligand 4 (CCL4) emerges as the most impactful feature affecting recovery concentration in the SHAP analysis, while it also shows a strong correlation within the correlation network (*r* = 0.57). In particular, IL‐6 contributes significantly to IL‐10 concentrations 1 h post exercise (Figure 1F), providing data‐driven confirmation of the IL‐6/IL‐10 interaction already described in the literature [8, 13] based on an experimental exercise trial with a broad immunological protein panel in humans. Additionally, monocyte chemoattractant protein‐3 (MCP‐3), IL‐10 itself during exercise, and IL‐5 are also among the five most important features of the SHAP analysis and appear to have a strong influence on IL‐10 concentration 1 h post exercise (Figure 1F). All figures of the significant proteins are available under https://sportsmedicine‐dortmund.shinyapps.io/beat.

In conclusion, we identified 44 of 92 immunological proteins displaying significant time‐dependent changes. Cluster analysis revealed distinct kinetic patterns, highlighting their dynamic temporal regulation and potential mechanistic interactions.

Even acute exercise of brief duration triggers effects on several immunological proteins, whereas longer exercise duration is associated with more pronounced increases [14]. Therefore, when exercise intensity remains constant, a longer exercise duration seems to promote stronger and more distinct immunological adaptations, which are reflected in greater changes in circulating protein levels.

## Author Contributions

N.J. and P.Z. conceptualized the study. T.W., S.T., D.W., and N.J. designed and performed the experiments. K.M. conducted outcome measurement. C.W. processed the data and conducted statistical and bioinformatic analyses. C.W., N.J., P.Z., and D.W. interpreted the data. C.W. and N.J. drafted the manuscript. All authors revised the manuscript draft and approved the final version of the paper. A.G. processed the data and conducted statistical and bioinformatic analyses and interpreted the data.

## Conflicts of Interest

The authors declare no conflicts of interest.

## Supporting information


**Table S1:** This Excel file contains two sheets. The first sheet lists all measured proteins with their abbreviations and full names. The second sheet presents the results of the linear mixed models (LMMs) for each analyte, including estimated fixed effects, standard errors, degrees of freedom (DF), *t*‐values, raw and adjusted *p*‐values. Significant effects (*p* < 0.05) are highlighted in bold.

## Data Availability

The data that support the findings of this study are openly available in Immune signature and exercise at https://sportsmedicine‐dortmund.shinyapps.io/beat. Further data are available at https://sportsmedicine‐dortmund.shinyapps.io/beat. The raw data are available upon request from the corresponding author.
